# Isolate-Dependent Growth, Virulence, and Cell Wall Composition in the Human Pathogen *Aspergillus fumigatus*

**DOI:** 10.1371/journal.pone.0100430

**Published:** 2014-06-19

**Authors:** Nansalmaa Amarsaikhan, Evan M. O’Dea, Angar Tsoggerel, Henry Owegi, Jordan Gillenwater, Steven P. Templeton

**Affiliations:** Department of Microbiology and Immunology, Indiana University School of Medicine – Terre Haute, Terre Haute, Indiana, United States of America; Geisel School of Medicine at Dartmouth, United States of America

## Abstract

The ubiquitous fungal pathogen *Aspergillus fumigatus* is a mediator of allergic sensitization and invasive disease in susceptible individuals. The significant genetic and phenotypic variability between and among clinical and environmental isolates are important considerations in host-pathogen studies of *A. fumigatus*-mediated disease. We observed decreased radial growth, rate of germination, and ability to establish colony growth in a single environmental isolate of *A. fumigatus*, Af5517, when compared to other clinical and environmental isolates. Af5517 also exhibited increased hyphal diameter and cell wall β-glucan and chitin content, with chitin most significantly increased. Morbidity, mortality, lung fungal burden, and tissue pathology were decreased in neutropenic Af5517-infected mice when compared to the clinical isolate Af293. Our results support previous findings that suggest a correlation between *in vitro* growth rates and *in viv*o virulence, and we propose that changes in cell wall composition may contribute to this phenotype.

## Introduction

*Aspergillus fumigatus* is a ubiquitous filamentous mold that is associated with pulmonary pathology in patients suffering from asthma, cystic fibrosis, and immune deficiencies [1]. In otherwise healthy individuals, inhalation of *A. fumigatus* conidia (asexual spores) has been associated with allergic sensitization and hypersensitivity pneumonitis [2], [3]. In patients with chronic lung inflammatory diseases such as asthma or cystic fibrosis, inhalation of *A. fumigatus* can lead to allergic bronchopulmonary aspergillosis (ABPA), which is marked by fungal persistence in the airways and increased inflammatory responses. However, the most severe disease occurs in neutropenic individuals or patients treated with immune suppressive drugs after hematopoietic stem cell or organ transplantation. These patients are susceptible to development of invasive aspergillosis (IA), a serious infection associated with a high mortality rate [1], [4].

Studies that attempt to identify virulence factors of *A. fumigatus* may be confounded by the extensive genetic and phenotypic variability observed between fungal isolates [5]. Sampling of health care centers reported a large diversity among clinical and environmental isolates in patients and in areas associated with patient care; in some instances changes in the environmental isolates that were sampled were seen over several months at the same location [6]–[11]. Although isolates may exhibit variability, only individual strains were able to be isolated from patients with aspergillosis [12]. Not surprisingly, when studied in experimental models, clinical isolates with higher *in vitro* growth rates exhibited increased virulence in mice when compared to slower growing isolates [13] or environmental isolates [14], [15]. Therefore, there is a correlation between isolate virulence and *in vitro* growth rates, although specific phenotypic differences that may play a role in this association have yet to be closely examined.

Through targeted mutation of *A. fumigatus* genes, numerous virulence factors have been identified [1], [16]–[19]. These include genes involved in thermotolerant growth, cell wall integrity, secretion of toxic metabolites, and the fungal response to environmental stress. To maintain a barrier of protection from the external environment, the cell wall of *A. fumigatus* contains α and β-glucans, chitin, galactomannan, melanin, and rodlet hydrophobins [19]–[21]. Not all of the genes that encode cell wall components are required for virulence in experimental invasive aspergillosis. For instance, deletion of the α-glucan encoding *ags1* or *ags2* had no effect on virulence, while mutation of *ags3* increased fungal disease [22]. Chitin, a polymer of N-acetylglucosamine that is covalently linked to β-glucan, is encoded by at least seven chitin synthase (*chs*) genes in *A. fumigatus* [23]. Deletion of individual *chs* genes did not alter fungal virulence in mice, though a double *chsC/G* mutant exhibited decreased growth and virulence [24]. Thus, fungal chitin synthesis is marked by redundancy, indicating the importance of this component to the growth and survival of *A. fumigatus*.

In this study, we examined phenotypic differences between two clinical and two environmental isolates of *A. fumigatus*. The two clinical strains, Af293 and Af13073, and one of the environmental strains (Af164), were similar with respect to *in vitro* radial growth, rate of germination, ability to establish colony growth, and cell wall chitin and β-glucan content. However, the environmental isolate Af5517 exhibited decreased radial, colony formation, and rate of germination along with increased hyphal diameter and cell wall chitin and β-glucan. Despite these differences, Af5517 was able to induce invasive aspergillosis in neutropenic mice, though with reduced virulence, lung inflammation, and *in vivo* fungal growth when compared to Af293. Thus, phenotypic differences may partly explain differences in virulence observed between clinical and environmental isolates of *A. fumigatus*.

## Results

### Decreased Radial Growth Rate and Colony Formation by the Environmental Isolate Af5517

**Figure 1:**
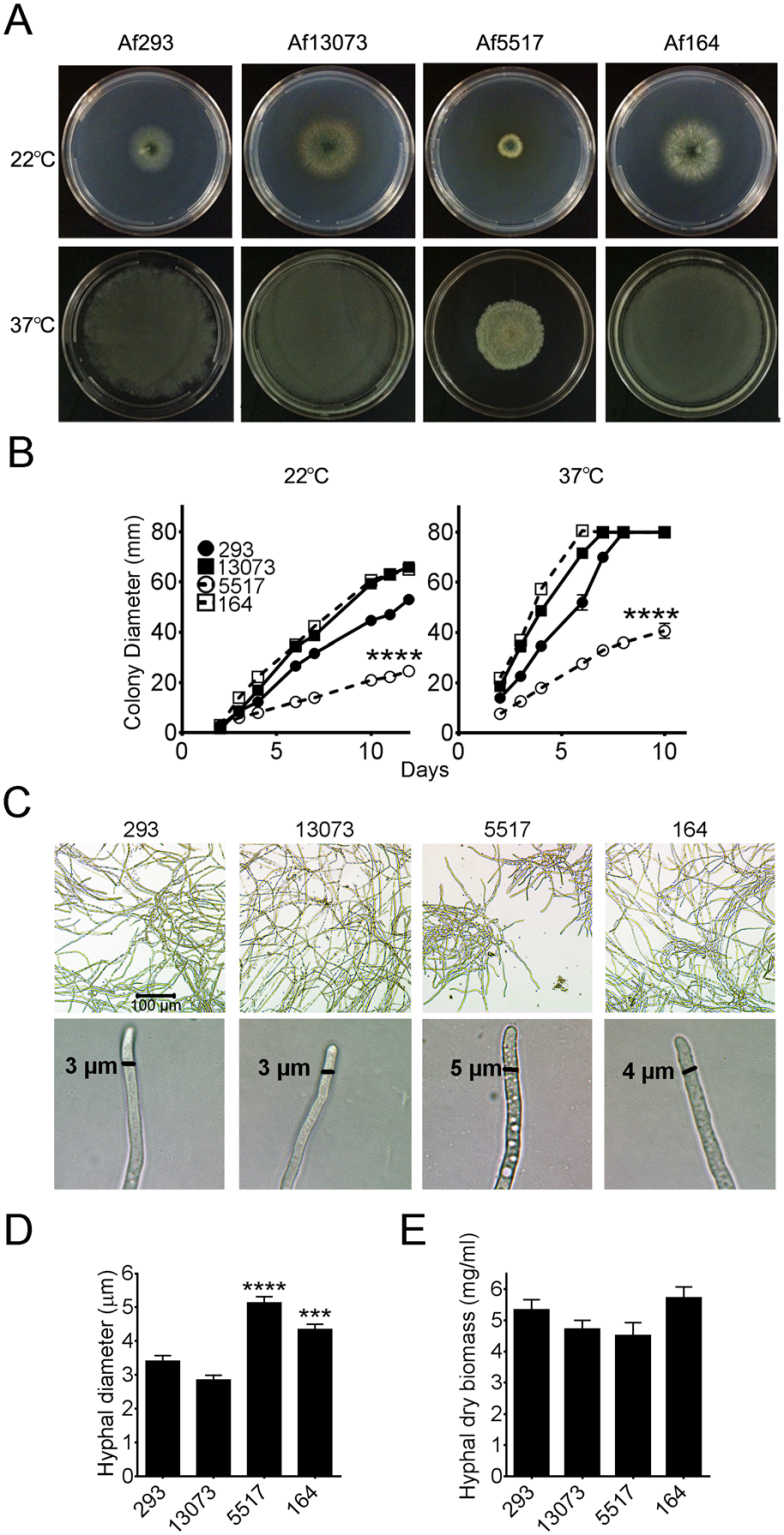
Decreased radial growth of *A. fumigatus* isolate Af5517. (**A**) Representative *Aspergillus* minimal media (AMM) plates 10 days after inoculation with 100 conidia of the indicated *A. fumigatus* isolates in triplicate. Plates were incubated at either 22°C or 37°C, as indicated. (**B**) Colony diameter during this incubation, measured daily. Clinical isolates are depicted with filled symbols connected by solid lines, while environmental isolates are open symbols connected by dotted lines. Growth of Af5517 was significantly different from all isolates after 4 days of growth. (**C**) Top panels, hyphal morphology of isolates after 24 hours growth in liquid culture at 37°C without shaking. Bottom panels, hyphal width was measured using SPOT Basic Software. (**D**) Summary of hyphal diameter measurements (n = 20–22/group). The diameters of Af5517 and Af164 hyphae were significantly increased when compared to Af293 and Af13073, and Af5517 diameter was increased compared to Af164 (p<0.01). (**E**) Hyphal mass accumulation of isolates after 24 hours growth in liquid culture with shaking. Data depict a summary of two experiments (n = 6/group). Each panel displayed is representative of two experiments. ***p<0.001, ****p<0.0001.

We were interested in the phenotypic differences between clinical and environmental isolates of *A. fumigatus*. Results of a previous study indicated that the *in vitro* growth rates of *A. fumigatus* isolates exhibited significant variation [13]. For our study, we screened two clinical and two environmental isolates of *A. fumigatus* for phenotypic differences (Table 1). The radial growth of each isolate over the course of 10–12 days at ambient (22°C) and physiological (37°C) temperatures was compared. The two clinical isolates, Af293 and Af13073 exhibited steady radial growth at both temperatures, as did the environmental isolate Af164 (Figure 1A, B). In contrast, isolate Af5517 exhibited a significantly reduced growth rate in comparison to all other isolates, reaching a colony diameter that was reduced by half by 10 days after inoculation. After 24 hours in liquid culture, Af5517 conidia formed smaller, yet denser hyphal aggregates when compared to the other isolates (Figure 1C, top panels). Furthermore, Af5517 and Af164 displayed more hyphal vacuoles and an increase in hyphal diameter (Figures 1C and 1D). Despite the differences in radial growth and hyphal morphology, Af5517 hyphae did not exhibit altered biomass accumulation (Figure 1E).

**Table 1:** List of *A. fumigatus* isolates used in this study.

*Source Name*	*Study Name*	*Source*	*Repository*
Af293	Af293	Clinical	Fungal Genetics Stock Center
ATCC13073	Af13073	Clinical	American Type Culture Collection
NRRL5517	Af5517	Environmental	U.S. Agricultural Research Service
NRRL164	Af164	Environmental	U.S. Agricultural Research Service

**Figure 2:**
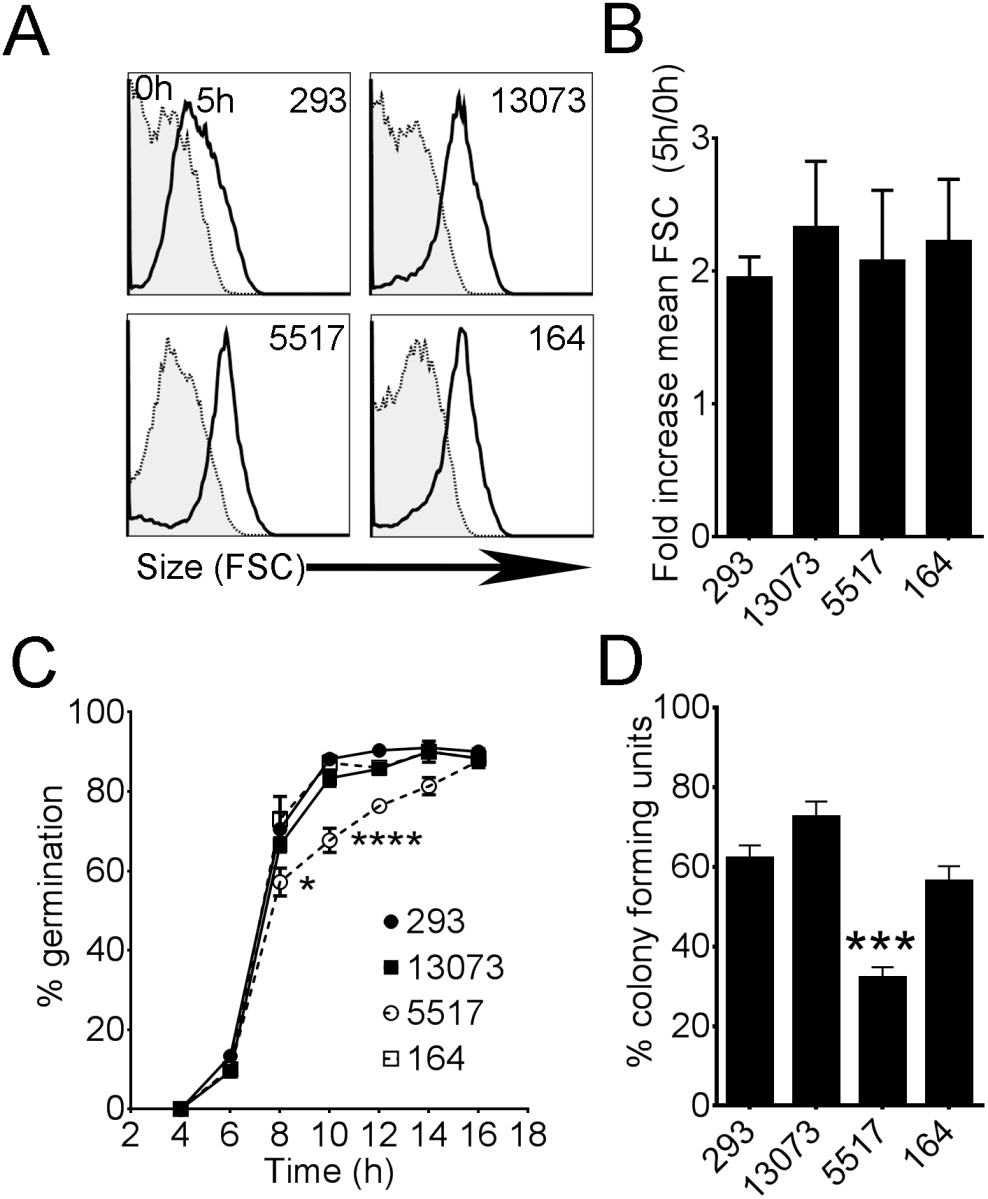
Decreased rate of germination and ability to establish colony growth by *A. fumigatus* isolate Af5517. (**A,B**) Flow cytometric analysis of conidial swelling in *A. fumigatus* isolates during 5 hours of incubation at 37°C. (**A**) Representative histograms from three experiments of forward scatter (FSC) from each isolate at 0 and 5 hours. (**B**) Increase in size (Conidial swelling) = FSC 5 h/FSC 0 h. Data are a summary of three experiments (n = 5). (**C**) Temporal quantification of germling formation by microscopic analysis. Summary of two experiments (n = 6). (**D**) Percent colony growth (CFU = colony forming unit), averaged from inoculations of 1000, 100, and 10 conidia, each in triplicate. Graphed data are a summary of two experiments. Decreased ability of Af5517 to establish colony growth was significantly different from all other isolates. ***p<0.001.

We also compared the ability of each isolate to initiate conidial swelling and germination and support colony growth by agar plate dilution and quantification of resulting colonies. By flow cytometric analysis of forward scatter (size), we observed that the fold increase in size (i.e. conidial swelling) of each isolate was equivalent after 5 hours of incubation in AMM at 37°C (Figures 2A and 2B). In contrast, by visual examination of germling formation, germination of Af5517 was reduced after 8 and 10 hours (Figure 2C). The ability of Af5517 conidia to support colony growth on solid agar was also reduced (Figure 2D). These results suggest that the ability of Af5517 conidia to form germlings and establish colony growth on solid media is defective. Furthermore, these differences do not appear to be due to decreased viability, as early conidial swelling appeared to be equivalent, the rate of germination was equivalent at 16 hours, and an increased inoculum of conidia did not result in increased radial growth in isolate Af5517 (Figure S1).

### Altered Cell Wall Composition in Isolate Af5517

**Figure 3:**
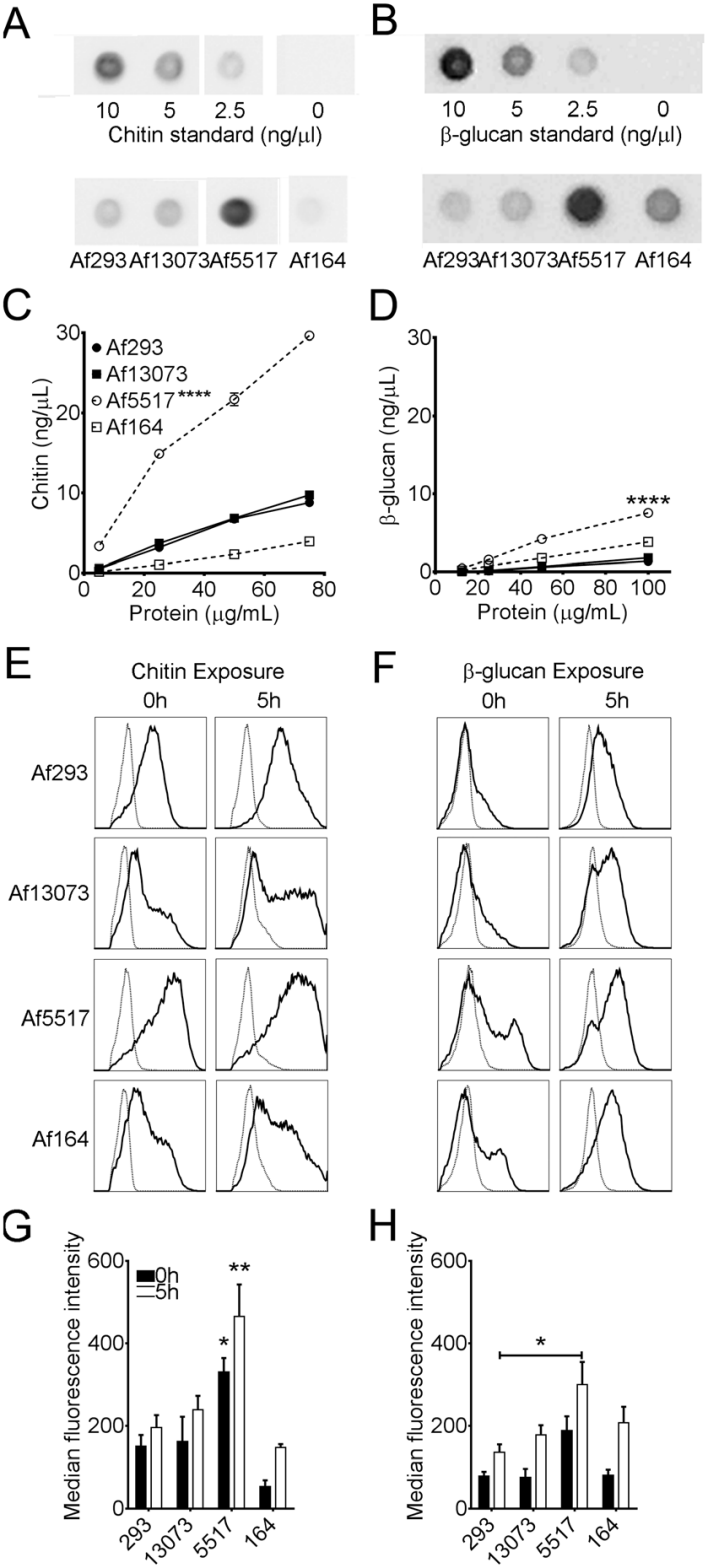
Increased cell wall chitin and β-glucan in *A. fumigatus* isolate Af5517. (**A,B**) Representative dot-blot images of processed *A. fumigatus* mycelial extracts and chitin (shrimp shell chitin) (**A**) or β-glucan (curdlan) (**B**) standards, probed with chitin binding probe or anti-(1,3)-β-glucan, respectively. Blots of mycelial extracts depict 75 µg (A) and 100 µg (B) of total protein of each isolates. (**C,D**) Chitin (**C**) or β-glucan (**D**) vs. total fungal protein in mycelial extracts as determined by dot blot assay. (**C**) Chitin was significantly increased compared to other isolates at 25, 50, or 75 µg of total protein, while β-glucan (**D**) was significant at 50 and 100 µg total protein. ****p<0.0001. (**A–D**) Panels are representative of two experiments. (**E,F**) Representative flow cytometric histograms of dormant (0 h) or swollen (5 h at 37°C) conidia of each isolate stained with the chitin binding wheat germ agglutinin (WGA, panel E) or anti-(1,3)-β-glucan (**F**). Negative controls are unstained conidia (**E**) or goat anti-mouse alexa-fluor 488 only (**F**) and depicted as dotted histograms. (**G,H**) Median fluorescence intensities of WGA (**G**) or anti-β-glucan (**H**) stained conidia after 0 or 5 hours incubation at 37°C. (**G**) Chitin exposure in Af5517 conidia was significantly increased in comparison will all other isolates after 0 and 5 hours incubation. *p<0.05. **p<0.01. Panels are a summary of three experiments.

Previous published reports have demonstrated altered growth upon mutation of cell wall components of *A. fumigatus*. More specifically, β-glucan and chitin contribute to the structural rigidity of the fungal cell wall, and changes in these components might affect radial growth [25]. In order to determine if the relative composition of β-glucan and chitin in the cell wall was different between isolates, we performed dot blot assays using a β-glucan-specific antibody or a chitin-binding probe [26]. In our assays, these reagents proved to be highly specific, as no cross-reactivity with other cell wall components was observed with the chitin binding probe or anti-β-glucan (Figure S2). When compared to the other isolates, the amount of chitin in mycelial extracts of Af5517 was markedly increased (Figure 3A, C). The relative amount of β-glucan was also increased in Af5517 mycelia (Figure 3B, D). Thus, the cell wall composition in Af5517 is altered when compared to other isolates.

In dormant conidia, inner cell wall components such as chitin and β-glucan are masked by an inert hydrophobic rodlet layer [27]. Upon swelling and germination, this layer is degraded and the underlying carbohydrate layers are exposed [27], [28]. Since chitin and β-glucan are immune modulatory [23], [29], increased exposure of these molecules may affect the ability of host cells to clear swelling or germinating conidia. Therefore, we stained conidia of each isolate with a chitin-specific probe, wheat germ agglutinin (WGA), or a β-(1,3)-glucan-specific antibody. We incubated these conidia in AMM for 5 hours, and compared the surface exposure of chitin and β-glucan by flow cytometry. After 0 and 5 hours incubation, Af5517 conidia displayed a higher level of chitin exposure when compared to the other isolates (Figures 3E, G). β-glucan exposure was more modestly increased in Af5517 conidia, and only with statistical significance after 5 hours and only in comparison to Af293 (Figures 3F, 3H). Thus, both chitin and β-glucan levels and surface exposure are increased in isolate Af5517, with a more marked increase in chitin.

### Decreased Lung Inflammation, Fungal Burden, and Virulence of Af5517 in Neutropenic Mice

**Figure 4:**
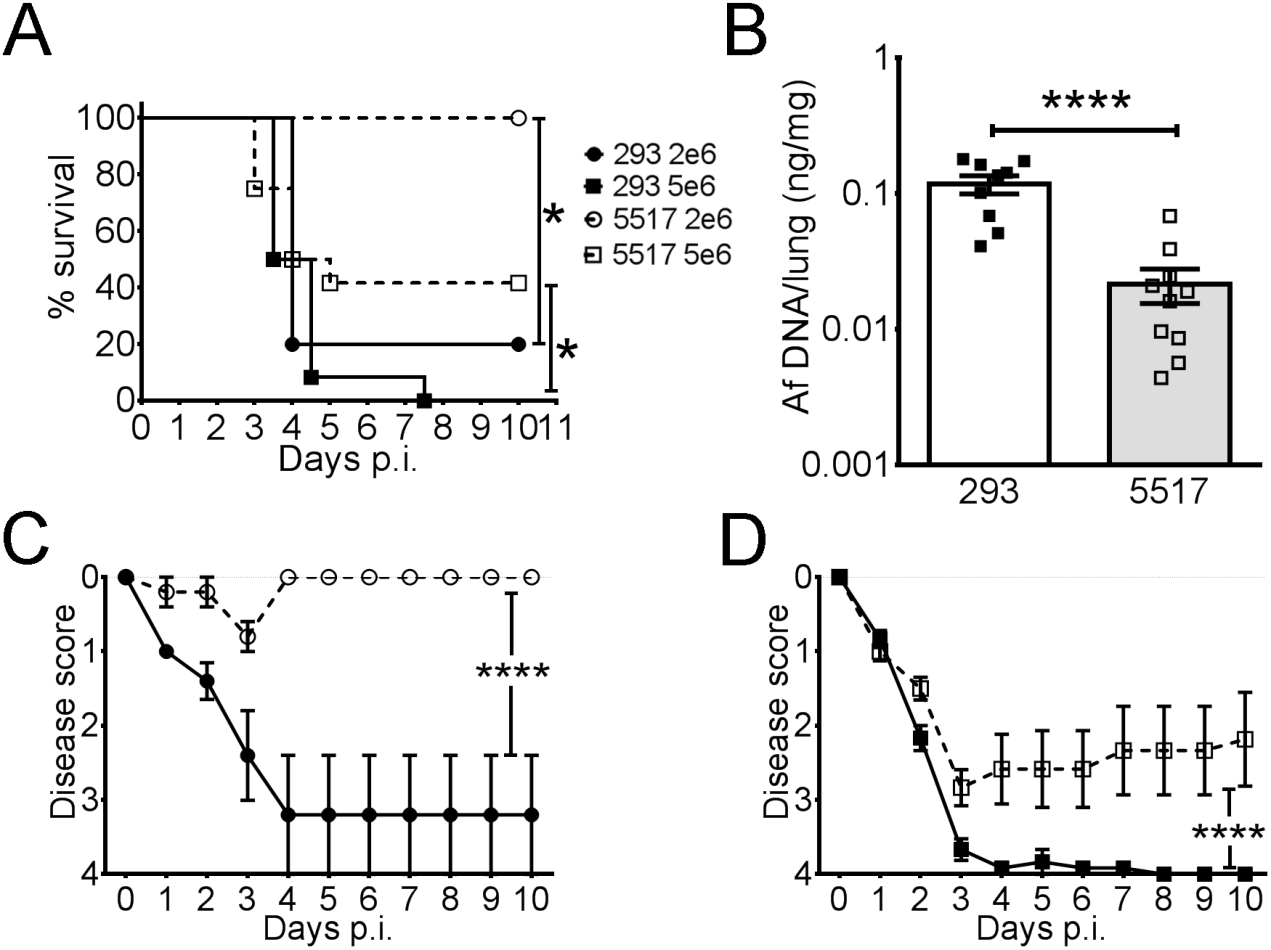
*A. fumigatus* isolate Af5517 is less virulent than Af293 in an animal model of pulmonary IA. Neutropenic BALB/c mice were infected with 2×10^6^ (circles) or 5×10^6^ (squares) conidia of isolates Af293 (closed symbols) or Af5517 (open symbols). (**A**) Survival curves depict death or moribund status for experimental animals over the course of the experiment. (**B**) Fungal burden of mice infected with 5×10^6^ conidia of Af293 (closed symbols) or Af5517 (open symbols) was determined by quantification of fungal 18S rDNA. (**C,D**) Disease scores of mice infected with 2×10^6^ (**C**) or 5×10^6^ (**D**) conidia. Mice were scored daily for progression of disease as described in Materials and Methods. Graphed data depicts the summary of two experiments with 5–8 mice per group in panels A, C, and D. *p<0.05. ****p<0.0001.

A previous study reported that the *in vitro* growth rates of isolates of *A. fumigatus* were proportional with the severity of disease in a mouse model of invasive aspergillosis [13]. Since the *in vitro* radial growth and colony formation of Af5517 were decreased in comparison with other isolates, we hypothesized that the virulence would also be decreased. Because the other isolates were phenotypically similar, and to limit the use of experimental animals, we therefore chose to more closely examine the virulence of Af5517 by infecting mice with doses of 2×10^6^ or 5×10^6^ Af293 or Af5517 conidia. Af293 was chosen for comparison because it has been well-characterized in genomic studies and is commonly used in murine models of infection and immunity [30]–[32]. Furthermore, in preliminary studies, we did not detect significant differences in virulence between Af293 and the other isolates used in this study, Af13073 and Af164 (data not shown). When compared to Af5517, Af293-infected mice exhibited severe disease with no survival at the 5×10^6^ dose, while at the 2×10^6^ dose mortality was slightly decreased (Figure 4A). In contrast, only mice infected with the higher dose of Af5517 had the potential to become moribund. The symptoms of clinical disease over the course of infection were also more severe in Af293-infected mice at both doses (Figure 4C, D). Furthermore, the lung fungal burden at day 3 post-infection was markedly lower in Af5517-infected mice (Figure 4B).

**Figure 5:**
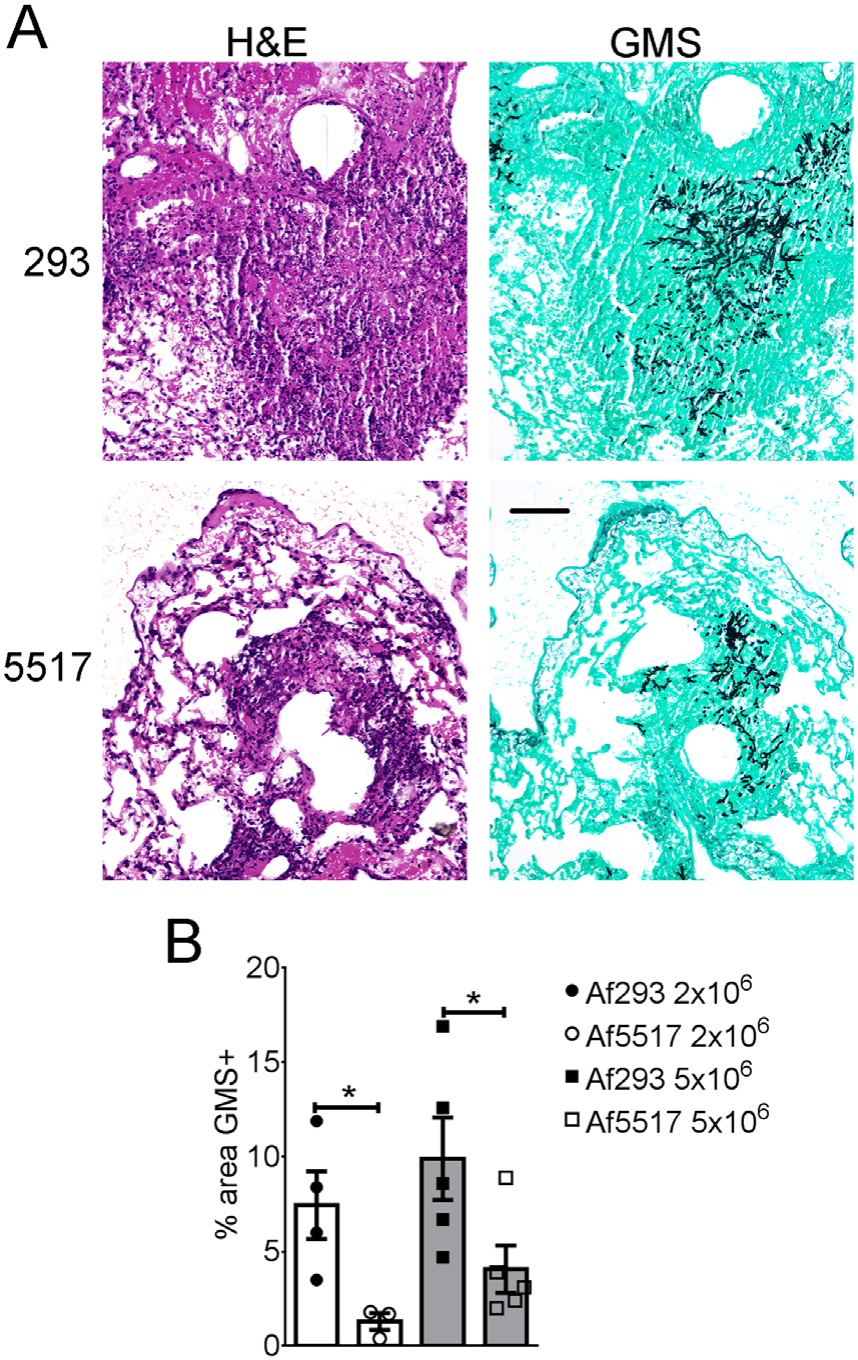
Histopathology of Af293 and Af5517 infection. Neutropenic BALB/c mice were infected with Af293 or Af5517 and sacrificed after 3 days for lung histological analysis. (**A**) Hematoxylin and Eosin (H&E)-stained sections (left panels) depict lung inflammation in mice infected with 5×10^6^ conidia. Adjacent Gomori’s Methanamine Silver (GMS)-stained sections (right panels) show areas of fungal growth. The black bar (bottom right panel) is equivalent to 100 µm. (**B**) GMS staining representing fungal growth was quantified in sections from mice infected with 2×10^6^ or 5×10^6^ conidia, with the mean of four representative fields displayed for each sample. *p<0.05.

In addition to the above factors, lung inflammation and *in vivo* fungal growth were also compared in histological sections from neutropenic mice infected with both isolates. Mice infected with Af293 displayed larger foci of inflammation and fungal growth that extended further into the lung parenchyma, whereas Af5517 infection and resultant inflammation was more bronchiocentric (Figure 5A). When lung fungal growth was quantified in Gomori’s Methanamine Silver (GMS) stained sections, areas of fungal growth were significantly larger in Af293-infected mice with both doses tested (Figure 5B). Therefore, Af5517 displayed a less virulent phenotype in neutropenic mice than Af293.

## Discussion

We observed that decreased radial growth of a single isolate, Af5517, was correlated with decreased virulence in a mouse model of invasive pulmonary aspergillosis. Although Af5517 conidia displayed a reduced ability to establish colony growth, our results suggest this is not due to decreased conidial viability, but rather due to limited and/or delayed growth after germination. We observed that conidial swelling was equivalent between isolates, and formation of germlings in Af5517 conidia appeared defective after 8 hours in liquid culture, reaching equivalent levels by 16 hours. Thus, the ability of *A. fumigatus* conidia to establish colony growth is not necessarily a direct measure of conidial viability.

In this study, we observed increased β-glucan and chitin content in the cell wall of the environmental isolate Af5517 when compared to the clinical isolates Af293 and Af13073 and the environmental isolate Af164. In *A. fumigatus*, chitin synthesis is controlled by at least 7 genes that appear to be redundant, though there is evidence that several of these genes control different aspects of cell-wall synthesis during growth and germination [23]. In some instances, β-glucan and/or chitin synthesis may be affected by regulatory networks that are activated in response to environmental stress. These stresses include contact with antifungal drugs or growth in hypoxic conditions [33], [34]. Other studies have shown that disruption of fungal genes such as the N-glycosylation-regulating gene Afstt3 increased cell-wall chitin via activation of the unfolded protein response (UPR), which is an ER stress-response [35]. It is interesting that both β-glucan and chitin content were increased in Af5517, since experimental evidence suggests that in some instances these components are reciprocally regulated. For example, chitin was increased in fungi grown in the presence of the β-glucan synthesis inhibitor caspofungin, while β-glucan was increased in the presence of the chitin synthesis-inhibiting nikkomycin Z [34]. Combined with our results, it appears that although reciprocal regulation is possible, it is not imperative. It is possible that in the isolate Af5517, multiple stress response regulatory genes may be constitutively activated, and this has resulted in alteration of cell wall content. Although chitin is a durable polymer of N-acetylglucosamine that possesses a high tensile strength that helps protect fungi from environmental stress [23], it is possible that an abundance of this rigid cell wall component could prove detrimental to fungal growth. Interestingly, Af5517 exhibited the largest hyphal diameter of all of the isolates examined, and it is likely that the altered cell wall composition accounts for this increase. Furthermore, our results suggest that optimal chitin expression may prevent abnormal growth rates exhibited by Af5517.

In addition to their roles as structural components of the fungal cell wall, β-glucan and chitin differentially stimulate host immune responses. Of these components, the response to β-glucan has been more extensively studied, though initial studies often used the fungal preparation zymosan, which also contains mannan and other immune-stimulating components [36]. Fungal β-glucan is recognized by the C-type lectin receptor Dectin-1 after degradation of the rodlet layer during germination results in nascent surface exposure [27], [29], [37], [38]. In our study, surface exposure of both chitin and β-glucan were increased in swollen Af5517 conidia. This altered expression could result in increased clearance of germinating Af5517 conidia, and thus might account for some of the observed decrease in virulence in Af5517-infected mice. Dectin-1 stimulation promotes phagocytosis, killing, and secretion of TNFα and CXCL2 in response to fungal particles [29], [39]. In murine pulmonary responses to *A. fumigatus*, dectin-1 mediated TNF-α production and provided protection from infection [40], [41]. Under hypoxic conditions, β-glucan and chitin expression were increased by *A. fumigatus*, resulting in increased macrophage and neutrophil fungal killing that was partly dependent on dectin-1 [33]. In contrast to studies of β-glucan, much less is known about chitin recognition by the immune system. A distinct chitin-binding receptor has yet to be identified, although TLR9, NOD2, and the mannose receptor were shown to interact with and be necessary for chitin-mediated macrophage IL-10 secretion [42]. Furthermore, chitin particles and chitin in fungal extracts mediated lung eosinophil recruitment in mice [43]–[45]. Eosinophils are effector cells associated with allergy and infection with helminths [46], intestinal parasites that, like fungi, express chitin [23]. In response to aspiration of Af5517 conidia, we have observed increased chitin-mediated airway eosinophil recruitment (O’Dea, E.M., Amarsaikhan, N., Li, H., Downey, J., Steele, E., Van Dyken, S.J., Locksley, R.M., Templeton, S.P. In press). Thus, the role of chitin expression and increased eosinophil recruitment in immune responses to *A. fumigatus* infection are the focus of our current and future investigation.

Although our study identified decreased growth in Af5517, other phenotypic differences between isolates could also contribute to decreased virulence and lung fungal burden. Numerous genes control the ability of *A. fumigatus* to support growth at physiological temperature, maintain cell wall integrity, process and uptake available nutrients, and respond to environmental stress [19]. Furthermore, several secreted toxins and fungal metabolites of *A. fumigatus* may interfere with immune-mediated host clearance, and these factors could be differentially expressed among clinical and environmental isolates [18]. In addition to the immune stimulatory chitin and β-glucan, expression of immune suppressive cell wall components such as galactomannan, melanin, and galactosaminogalactan might also differ between *A. fumigatus* isolates [47]–[49]. Results of a recent study suggest that immune responses to wild-type and mutant *A. fumigatus* strains also exhibit considerable variability [50]. It will thus be of interest to determine if these differences are driven by differential expression of immune-modulating cell wall components or other factors required for the growth and metabolism of *A. fumigatus* within host tissue. In order to completely understand the mechanisms of virulence and immune modulation by *A. fumigatus* isolates, extensive comparative analysis of genomic and proteomic differences between fungal isolates will likely be required.

In summary, we observed a decreased *in vitro* growth rate, rate of germination, and decreased colony formation along with increased hyphal diameter characterized by increased chitin and β-glucan in a single environmental isolate, Af5517, in comparison to other clinical and environmental isolates. In addition to these phenotypic differences, the virulence of Af5517 was also decreased in neutropenic mice. When considered together, the results of this and other studies support a correlation between *in vitro* growth rates and *in vivo* virulence [13]–[15]. Furthermore, alteration of cell-wall composition might be a contributing factor to this phenotype. However, more isolates that concurrently display slower growth, altered cell wall composition, and reduced virulence will need to be identified in order to conclusively define the relationships between these factors. In future studies, comparative analysis of fungal gene expression in these isolates will be necessary to identify candidate pathways responsible for associated changes in structure, growth, and virulence of this ubiquitous human pathogen.

## Materials and Methods

### Comparison of Morphology, Radial Growth, and Rates of Germination in *A. fumigatus* Isolates

Fungal isolates were obtained from the specified sources (Table 1). Fungi were grown on malt extract agar (MEA) plates at room temperature (RT) unless specified otherwise, and fungal conidia were isolated from plates using glass beads (BioSpec), and further separated from the beads by adding sterile PBS as previously described [51]. To compare radial growth, conidia were collected in sterile water, counted on a hemacytometer, and then diluted to 100 conidia/µL. A volume of 1 µL was pipetted onto the center of *Aspergillus* minimal media agar plates [52] and radial growth was measured daily for thirteen days. For microscopic examination of hyphal morphology, conidia were inoculated on coverslips immersed in 2 ml AMM overnight at 37°C. Coverslips were removed and washed with PBS and inverted on glass slides for microscopic analysis. To compare rates of germination, a previously described method was used, with slight modifications [53]. Briefly, conidia were incubated in 5×10^6^ conidia/ml in AMM at 37°C with shaking at 200 rpm. Sampling was performed at 4, 6, 8, 10, 12, 14, and 16 hours and observed under light microscopy at 400x magnification. 24 hour-germinated samples were lyophilized for total biomass measurement or were analyzed for comparison of hyphal morphology by microscopy. Germination rates were reported as percent of germ tube forming conidia per random 100 conidia count. To compare the ability of fungal isolates to establish colony growth, conidia were collected in sterile water with 0.05% Tween 20, very lightly sonicated to disperse clumps, counted on a hemacytometer, and diluted to three concentrations: 1000, 100, and 10 conidia/mL. A volume of 1 mL of each suspension was inoculated in triplicate on MEA plates, and colonies were counted after a 72 hour incubation at 37°C. For the described experiments, all samples were prepared and data acquired in triplicate.

### Comparison of Cell Wall Chitin and β-Glucan Content in *A. fumigatus* Isolates

To compare cell wall chitin and β-glucan content using a previously described dot blot method [44], isolates were cultured in malt extract broth for 5 days at room temperature with shaking at 100rpm. The hyphae were then lyophilized, bead-beaten, and suspended in sterile water with 0.05% Tween 20. Fungal protein concentrations were standardized using Pierce BCA Protein Assay kit (Fisher), and then the samples were diluted into four separate concentrations to ensure results within measurable range. Each sample was lightly sonicated to disperse clumps, then 1 µL was immediately dot-blotted in triplicate onto a nitrocellulose membrane (GE Healthcare). Shrimp shell chitin (Sigma-Aldrich) and curdlan (Wako) were used as positive controls. The membranes were dried at room temperature (RT), blocked in Tris buffered saline with 0.05% Tween 20 (TBST) with 5% nonfat dry milk, then incubated for 16–24 hours at 4°C with gentle rocking in TBST with 1% nonfat dry milk and either anti-(1–3)-β-glucan antibody (Biosupplies) or a probe containing a chitin-binding domain conjugated to FITC (expression clone provided by Yinhua Zhang, New England Biologicals) [54]. After washing with TBST, the membranes were incubated for 45 min at RT with gentle rocking in either HRP anti-mouse IgG (Jackson ImmunoResearch) or HRP anti-fluorescein (Invitrogen). After a final wash in TBST, membranes were developed with SuperSignal West Femto Substrate ECL kit (Fisher) and images were captured using a ChemiDoc-IT imaging system and analyzed with VisionWorks software (UVP). To quantify chitin and β-glucan exposure using a similar method that was previously described [55], conidia were kept dormant or allowed to germinate for 5 hours in AMM with gentle rocking, then stained with a 1∶500 dilution of the chitin-binding wheat germ agglutinin-APC (Invitrogen) or 1∶250 dilution of anti-β-glucan (Biosupplies) for 30 minutes on ice in the dark. For β-glucan detection, a 1∶500 dilution of a secondary goat-anti mouse Alexa-Fluor 488 (Invitrogen) was added after washing for a second 30 minute incubation. Stained conidia were analyzed for surface staining or changes in forward scatter (size) on a Guava EasyCyte 8 HT flow cytometer (EMD Millipore).

### Comparison of Fungal Virulence

BALB/c mice were obtained from Harlan Laboratories and bred in an AALAC accredited animal facility. Mice were used in experiments at 7–10 weeks of age. All animal procedures were approved by the Indiana State University Animal Care and Use Committee. For examination of virulence, mice involuntarily aspirated 50 uL sterile PBS containing 2×10^6^ or 5×10^6^ conidia of either Af293 or Af5517. Relative to this aspiration, neutrophils were depleted via IP injection of 0.5 mg anti-mouse-Ly-6G antibody (clone 1A8; BioXCell) one day before and after. Following aspiration, mice were scored daily for morbidity and mortality up to ten days. Morbidity was scored from 0 to 4 as follows: 0) healthy 1) minimal disease (e.g. ruffled fur), 2) moderate disease (e.g. ungroomed, hunched), 3) severe disease (e.g. severely hunched, changes in eye color, low motility), and 4) moribund/deceased. Mice that became moribund and received a score of 4 were sacrificed. For histological comparison, mice were sacrificed 3 days post-infection, and lungs were perfused with 5 ml of saline followed by 5 ml of 10% formalin buffered saline, followed by inflation using a tracheal catheter with 1 ml of formalin buffered saline. Lungs were then removed and allowed to fix overnight in formalin buffered saline, followed by tissue embedding processing and staining of sections with hematoxylin and eosin and Gomori’s methanamine silver stains. All histological sample processing and staining was performed by the Terre Haute Regional Hospital pathology laboratory

### Fungal Burden Assay

To quantify fungal burden, mice were sacrificed three days after infection and lungs were collected and rapidly frozen in liquid nitrogen. Genomic DNA was extracted from 50–100 mg freeze dried, homogenized whole lung tissue using a previously described DNA extraction buffer for *Aspergillus* nucleic acids with subsequent phenol/chloroform extraction. A total of 500 ng genomic DNA was used for quantitative PCR to determine the fungal DNA content [56], [57]. A qPCR fungal burden assay was performed to determine the amount of fungal 18S rDNA in lung extracts with 18S rDNA primer and probe sets with a modified probe quencher (5′-/56-FAM/AGC CAG CGG/ZEN/CCC GCA AAT G/3IABkFQ/-3′) [56], [58]. A standard curve was prepared from Af293 genomic DNA and used to calculate the concentration of fungal DNA in each sample, with uninfected mouse lungs analyzed to confirm absence of contamination. Samples were amplified in duplicate with at least five technical replicates. The qPCR reaction was performed in Agilent Mx3005P with MxPro Software (Agilent Technologies). Ct values were used to calculate the corresponding fungal DNA content in the lung tissue and the fungal burden was reported as ng fungal DNA per mg of total lung DNA.

### Data Analysis

Data analysis and resulting graphs were performed and prepared with Prism software (GraphPad). For statistical comparison of two groups, unpaired t tests were used. For multiple groups, one- or two-way ANOVA were used with Tukey’s or Holm-Sidak’s test for multiple comparisons, respectively. To compare survival or disease scores between groups, the Mantel-Cox log-rank test was used. The hyphal diameter of each isolate was measured using SPOT Basic Software (Diagnostic Instruments, Inc). For quantification of fungal growth on histological sections, the mean of four representative fields at 100x magnification of GMS+ staining for each sample was calculated using ImageJ software (National Institutes of Health).

## Supporting Information

Figure S1 **Radial growth of Af293 and Af5517 with increased inocula of Af5517 conidia.** AMM plates were centrally inoculated with the indicated isolate and inoculum, and allowed to grow for 7 days, with the resulting diameter of growth measured. Data displayed are representative of two experiments.
(TIF)

Figure S2 **Specificity of anti-β-glucan antibody and chitin binding probe for cell wall components of** ***Aspergillus fumigatus*** **.** Shrimp shell chitin (Ch), curdlan (βg), and locust bean gum galactomannan (Gm) were blotted together on three separate membranes that were probed with chitin binding probe, anti-β-glucan, and ConA, respectively. Data displayed are representative of samples blotted in triplicate that gave similar results.
(TIF)
