# Supplementary figures and images for: Isolate-Dependent Growth, Virulence, and Cell Wall Composition in the Human Pathogen Aspergillus fumigatus
Source: PLoS One. 2014 Jun 19;9(6):e100430. doi: 10.1371/journal.pone.0100430 (PMC4063936; doi:10.1371/journal.pone.0100430)

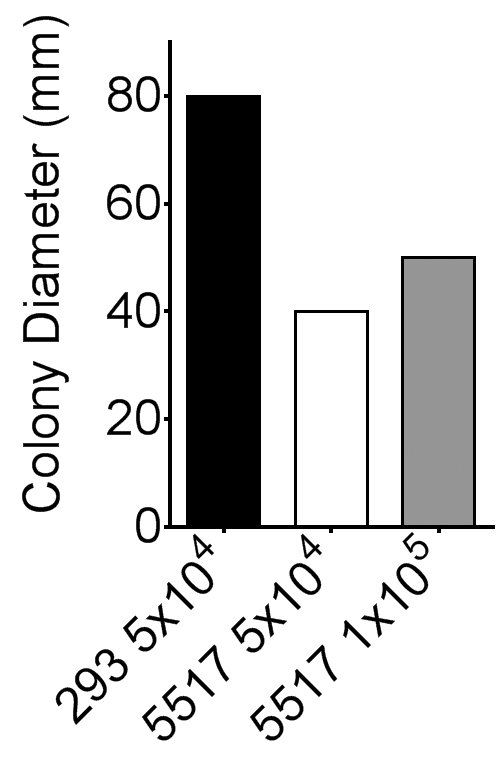

Supplement: Figure S1 — Radial growth of Af293 and Af5517 with increased inocula of Af5517 conidia. AMM plates were centrally inoculated with the indicated isolate and inoculum, and allowed to grow for 7 days, with the resulting diameter of growth measured. Data displayed are representative of two experiments. (TIF) [file pone.0100430.s001.tif]

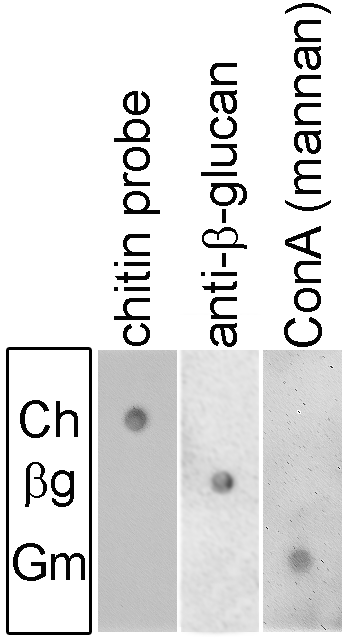

Supplement: Figure S2 — Specificity of anti-β-glucan antibody and chitin binding probe for cell wall components of Aspergillus fumigatus . Shrimp shell chitin (Ch), curdlan (βg), and locust bean gum galactomannan (Gm) were blotted together on three separate membranes that were probed with chitin binding probe, anti-β-glucan, and ConA, respectively. Data displayed are representative of samples blotted in triplicate that gave similar results. (TIF) [file pone.0100430.s002.tif]
